# Postpartum Depression at a Tertiary Care Hospital in Saudi Arabia: Prevalence and Associated Factors

**DOI:** 10.7759/cureus.37758

**Published:** 2023-04-18

**Authors:** Mohammed A Aljaffer, Ahmad H Almadani, Afnan A Almustafa, Ghaida Al Musma, Lama I Al Musallam, Maha Z Alamri, Reema Alageel, Shirin H Alokayli

**Affiliations:** 1 Department of Psychiatry, College of Medicine, King Saud University, Riyadh, SAU; 2 College of Medicine, King Saud University, Riyadh, SAU

**Keywords:** tertiary care hospital, saudi arabia, edinburgh postnatal depression scale, pregnancy, postpartum depression

## Abstract

Objectives

Postpartum depression (PPD) is a significant health concern associated with several risk factors. This study aims to assess the prevalence of PPD and its related factors in a tertiary care hospital in Riyadh, Saudi Arabia, specifically King Khalid University Hospital (KKUH).

Methods

A cross-sectional study was conducted of 187 females aged 18 to 50 years old who gave birth at KKUH. Data were collected from the same participants at two stages using the same questionnaire, which consisted of the Edinburgh Postnatal Depression Scale (EPDS) and demographic questions. In the first stage, the participants were selected randomly. The second stage included participants who scored less than 9 on the EPDS in the first stage and were asked to retake the questionnaire four weeks later.

Results

The prevalence of PPD found in this study was 50.3%, which is higher than in other studies that have been conducted in the country. Furthermore, factors such as sleep disturbances (p = 0.005), loss of interest in daily activities (p = 0.031), mood swings (p = 0.021), frequent bouts of sadness (p < 0.0001), and frustration or worry (p < 0.0001) were all found to significantly increase the risk of PPD.

Conclusion

This study demonstrates a high prevalence of PPD in women who delivered at KKUH. More studies with a more rigorous methodology are warranted.

## Introduction

The transition into motherhood typically requires significant psychological, social, and physical adjustments [1]. Caring for an infant is a highly demanding task, but new mothers are likely to demonstrate dedication in caring for their babies when they feel fulfilled in their role as mothers [1]. The mother-infant relationship is based on a strong connection that begins immediately after giving birth and grows afterward [2], and any disturbance to this connection may alter the baby’s development [3].

Mental illnesses, including depression, can take various forms. One common form of depression is postpartum depression (PPD). PPD is a form of major depressive disorder that begins within four weeks after delivery [2,4]. The symptoms of PPD include depressed mood, loss of interest, inability to feel pleasure, sleep and appetite disturbances, impairment of concentration, fatigue, feelings of guilt or worthlessness, and suicidal thoughts [3,5].

PPD can have several negative consequences on mothers [6]. Although psychological disorders, including depression, are fairly common during the postpartum period, both new and expecting mothers may be less inclined to seek care for common psychological disorders, as it has been found that the postpartum period is associated with lower rates of healthcare-seeking behaviors [7].

The prevalence of PPD is estimated to be 17.22% worldwide [8]. In 2021, a cross-sectional study of 385 patients in Mexico found the prevalence of PPD to be 16% [9]. In 2022 in Seoul, South Korea, a study examined 80,116 women who participated in the Seoul Healthy First Step Project in South Korea in 2013. The prevalence of PPD in this study was estimated to be 24.3% [10].

In Saudi Arabia, a study in Riyadh city in 2020 estimated the prevalence of PPD to be 38.50% [11]. Another study in Jeddah city in Saudi Arabia identified the prevalence of PPD to be 20.9% [12]. A third study, which was conducted in Al-Madinah city, found the prevalence of PPD to be 31.68% [13]. A cross-sectional study published in 2022 estimated the prevalence of PPD in Port Said, Egypt. This study examined 540 women at 10 weeks into the postpartum period using the Arabic version of the Edinburgh Postnatal Depression Scale (EPDS) as an assessment tool. The prevalence of PPD in this study was found to be 24.4% [14].

PPD has been linked to various factors. For instance, international studies found that young age, single parenthood [10], delivery complications and husband support during the pregnancy period [9], report of a health problem during the last pregnancy, perceived exposure to a significant number of life stressors [15], poor satisfaction in relationships with the husband and family [16], and lack of financial and social support [17] are all associated with PPD.

Studies in Saudi Arabia have also linked PPD to various factors. Factors such as recent stressful life events [11], mode of delivery [18], history of depression, attitude toward pregnancy [12], negative delivery experience, and unplanned cesarean section (CS) were all found to increase the risk of developing PPD. In contrast, factors such as income status, family support [19], and spousal support [18] were found to decrease the possibility of developing PPD.

Given the significance of this topic, this study aims to assess the prevalence of PPD and its associated factors in a tertiary care hospital in Riyadh City, Saudi Arabia.

## Materials and methods

Study design and place

This cross-sectional study aims to estimate the prevalence of PPD at King Khalid University Hospital (KKUH) in Riyadh City, Saudi Arabia. In addition, the study aims to explore the associated risk factors and their correlation with PPD.

Study variables

The study variables include the age, type of delivery, presence or absence of delivery complications, baby’s gender, social support, and previous history of depression or depressive symptoms. The outcome variable is depression.

Study subjects and data collection

Participants included 187 females who delivered at KKUH between November 2019 and April 2022. Eligible participants were postpartum mothers between 18 and 50 years of age. Women who had a vaginal delivery and those who had a CS were both included in the study. Any females under the age of 18 or over the age of 50 were excluded from the study. Informed consent was obtained from all participants. The sample size was calculated as N = (Z) 2 P (1-P)/D 2, with Z = 1.96 for the 95% confidence interval, p = 0.33, and D = 0.07. The sample size, therefore, should be 188 participants. The data were collected from the same participants in two stages using a questionnaire that was divided into two parts: the EPDS and demographic questions that included the mother’s age, type of delivery, presence or absence of delivery complications, the baby’s gender, social support, and previous history of depression or depressive symptom.

EPDS is a screening tool that includes a set of 10 questions used to identify women who may have PPD, with a score ranging from 0 to 30 [20]. The sensitivity of this scale is 100%, and its specificity is 98.13% [21]. In this study, participants were given either the Arabic or English version of the EPDS, according to their language preference. A score of 9 was considered the cutoff in this study, at which point the participant was considered to have PPD.

The participants were chosen at random and interviewed in the first week of the postpartum period at the Ob-Gyn ward. Participants who had a score of 9 or higher (minimum = 0, maximum = 30) were considered to have PPD and were not included in the second stage. Participants who scored less than 9 in the first stage were invited to re-complete the questionnaire four weeks after the delivery date either by phone or online questionnaire.

Statistical methods of analysis and ethical issues

A statistical analysis was performed using the Statistical Package for the Social Sciences (SPSS) version 24.0 software (SPSS Inc., Armonk, NY, USA) [22]. We calculated the frequencies and percentages of all nominal variables and the mean ± SD (standard deviation) for numerical variables. We then compared the total score from the first questionnaire with the total score from the second questionnaire using a paired t-test. We also used a chi-square test or Fisher’s exact test to compare the two groups of patients (patients with higher scores [more than 8] and those with lower scores [less than or equal to 8]). Results were considered significant when p-values were less than 0.05.

This study was approved by the Institutional Review Board (IRB) of the College of Medicine at King Saud University (project No. E-19-4453). The participants provided informed consent before participating. The participants were informed that they had the right to withdraw at any time without any obligations. Participants who scored 9 or above on the EPDS were counselled by the primary investigator, and they were directed to seek appropriate treatment when needed.

## Results

The ages of the 187 mothers who participated in this study ranged from 19 to 47 years, with SD = 5.2004. The distribution of the main demographic characteristics and their likelihood of developing PPD is illustrated in Table 1.

**Table 1:** Association between demographic characteristics and postpartum depression. SVD, spontaneous vaginal delivery; CS, cesarean section

Depression symptoms		Frequency	Group		P-value
			Likely to be depressed, n (%)	Unlikely to be depressed, n (%)	
Gender of baby	Male	101	51 (54.3 %)	50 (53.8 %)	0.946
	Female	86	43 (45.7 %)	43 (46.2 %)	
First baby	Yes	52	25 (27.5 %)	27 (31.8 %)	0.533
	No	124	66 (72.5 %)	58 (68.2 %)	
Type of delivery	SVD, normal delivery	127	66 (70.2 %)	61 (66.3 %)	0.567
	CS	59	28 (29.8 %)	31 (33.7 %)	
Complications during or after birth	Yes	32	16 (17.0 %)	16 (17.2 %)	0.973
	No	155	78 (83.0 %)	77 (82.8 %)	
Social support (psychological, moral)	Yes	179	89 (94.7 %)	90 (96.8 %)	0.366
	No	8	5 (5.3 %)	3 (3.2 %)	
History of depression	Yes	7	5 (5.3 %)	2 (2.2 %)	0.227
	No	180	89 (94.7 %)	91 (97.8 %)	

Approximately 54% (N = 101) of the participants delivered a male newborn, which represents 54.3% of the participants with high EPDS scores and 53.8% of the participants with low EPDS scores. In comparison, 46% of the total participants delivered a female baby. Of these participants, 50% were likely to be depressed, comprising 45.7% of participants with high EPDS scores and 46.2% of participants with low EPDS scores (P = 0.946).

Only 52 (29.5%) of the 176 participants were mothers for the first time. Of these, 25 were likely to have PPD, representing 27.5% of the participants with high EPDS scores, and 27 were not likely to have PPD, representing 31.8% of the participants with low EPDS scores. The remaining 124 participants had a baby who is not their first child, comprising most of the study’s sample (70.5%). Of these, 66 were likely to have PPD, representing 72.5% of the participants with high EPDS scores, while 58 were not likely to have PPD, representing 68.2% of the participants with low EPDS scores (P = 0.533).

Of the participants, 68.3% (N = 127) had a spontaneous vaginal delivery, representing 70.2% of the participants with high EPDS scores and 66.3% of the participants with low EPDS scores, respectively. In comparison, women who had a CS were the minority in the study sample (n = 59). These women comprised 29.8% of the participants with high EPDS scores and 33.7% of the participants with low EPD scores (P = 0.567).

Of the participants, 32 had issues during or after labor. Half (N = 16) were likely to have PPD, accounting for 17% of the participants with high EPDS scores, while the other half were unlikely to have PPD, representing 17.2% of participants with low EPDS scores.

Most of the participants did not have any issues during or after giving birth (82.9%, N = 155). Of these, 78 were likely to have PPD, representing 83% of the participants with high EPDS scores. In comparison, 77 were unlikely to have PPD, representing 82.8% of the participants with low EPDS scores (P = 0.973).

When the participants were asked whether they had received social support from people around them, the majority (95.7%, N = 179) answered yes. These participants represented 94.7% of the participants with high EPDS scores and 96.8% of the participants with low EPDS scores. The remaining eight participants who did not receive social support (4.3% of the total study sample) comprised 5.3% of the participants with high EPDS scores and 3.2% of the participants with low EPDS scores (P = 0.366).

Only 3.7% (N = 7) of the participants had a history of depression. Of these, five were likely to have PPD (5.3% of the participants with high EPDS scores), and two were unlikely to have PPD (3.2% of the participants with low EPDS scores). In comparison, the remaining 180 participants did not have any past history of depression. Of these, 89 were likely to have PPD (94.7% of the participants with high EPDS scores), and 91 were unlikely to have PPD (97.8% of the participants with low EPDS scores) (P = 0.227).

None of the demographic variables (gender of the baby, whether the baby was the first child, type of delivery, if there were any complications, whether or not they had social support, and if they had a history of depression) were statistically significant with an association to PPD.

The survey questions also asked regarding depressive symptoms either before or after pregnancy (Table 2). Among the study participants, 65.8% (n = 123) had sleep disturbances, 71 of which were likely to have PPD (75%.5 of the participants with high EPDS scores) (P = 0.005, OR = 2.434, 95% CI = 1.305-4.54). Among the participants, 21.9% (N = 41) felt guilty or remorseful. Of these, 26 were likely to have PPD (27.7% of participants with high EPDS scores) (OR = 1.988, 95% CI = 0.974-4.06). Among the participants, 51.9% (N = 97) experienced weight changes, 50 of which were likely to have PPD (53.2% of the participants with high EPDS scores) (OR > 1). When the participants were asked regarding the performance of their typical daily activities, 42.2% (N =79) answered that they had lost interest in doing any of their daily tasks. Of these, 47 were likely to have PPD (50% of the participants with high EPDS scores) (P = 0.031, OR = 1.906, 95% CI = 1.058-.434). Among the participants, 71.1% (N = 133) had experienced mood changes during or before pregnancy. Of these, the majority (N = 74) were likely to have PPD (78.7% of the participants with high EPDS scores) (P = 0.021, OR = 2.132, 95% CI = 1.113-4.083). When the participants were asked whether they had gone through any bouts of sadness during or before their pregnancy, 44.9% (n = 84) responded yes. Of these, 56 were likely to have PPD (59.6% of the participants with high EPDS scores) (P < 0.0001, OR = 3.421, 95% CI = 1.868-6.265). Approximately half of the participants (50.8%, n = 95) have experienced frustration or worry, and most (N = 60) of these participants are likely to have PPD (63.8% of the participants with high EPDS scores) (P < 0.0001, OR = 2.924, 95% CI = 1.614-5.297). Only 9.1% (N = 17) of the participants have not had any of the previous symptoms of depression before or during pregnancy, and of these, only seven were likely to have PPD (7.4% of the participants with high EPDS scores) (95% CI = 0.243-1.837).

**Table 2:** Association between symptoms of depression before or during pregnancy and postpartum depression.

Depression symptoms			Group		P-value	OR	95% CI	
			Likely to be depressed, n (%)	Unlikely to be depressed, n (%)			Lower	Upper
Sleep disturbances	Yes	123	71 (75.5)	52 (55.9)	0.005	2.434	1.305	4.54
	No	64	23 (24.5)	41 (44.1)				
Feeling remorse	Yes	41	26 (27.7)	15 (16.1)	0.057	1.988	0.974	4.06
	No	146	68 (72.3)	78 (83.9)				
Weight loss or gain	Yes	97	50 (53.2)	47 (50.5)	0.716	1.112	0.627	1.974
	No	90	44 (46.8)	46 (49.5)				
Loss of interest in daily activities	Yes	79	47 (50.0)	32 (34.4)	0.031	1.906	1.058	3.434
	No	108	47 (50.0)	61 (65.6)				
Mood swings	Yes	133	74 (78.7)	59 (63.4)	0.021	2.132	1.113	4.083
	No	54	20 (21.3)	34 (36.6)				
Frequent bouts of sadness	Yes	84	56 (59.6)	28 (30.1)	<0.0001	3.421	1.868	6.265
	No	103	38 (40.4)	65 (69.9)				
Frustration or anxiety	Yes	95	60 (63.8)	35 (37.6)	<0.0001	2.924	1.614	5.297
	No	92	34 (36.2)	58 (62.4)				
All symptoms	Yes	7	6 (6.4)	1 (1.1)	0.061	6.273	0.740	53.159
	No	180	88 (93.6)	92 (98.9)				
No symptoms	Yes	17	7 (7.4)	10 (10.8)	0.432	0.668	0.243	1.837
	No	170	87 (92.6)	83 (89.2)				

Concerning the prevalence of PPD among the participants in this study, approximately half of the postpartum women were found to have PPD (50.3%, n = 94). When comparing to the mean (paired samples) of the total EPDS scores of the participants who had lower scores on the first questionnaire (n = 69, mean = 4.64) and then had a follow-up by phone call (n = 69, mean = 6.26), the correlation between the first total scores and the second total scores was -0.033 (P = 0.785), and the mean (the difference between the first and second total scores) was -1.623 (95% CI = -3.006 to -2.41; Sig (two-tailed) = 0.022 [<0.05]).

## Discussion

This research focused on the prevalence of PPD and its associated factors among women who gave birth at KKUH using the EPDS. The prevalence of PPD reported in this study is approximately half of the overall participants (50.3%, N = 94), which is higher than in other studies conducted in the country, reporting 20.9% in Jeddah city and 31.68% in Al-Madinah city [12,13]. Moreover, it is higher than the prevalence that was reported by the World Health Organization (WHO) in 2019, which indicated that more than 10% of women globally and around 20% of women in developing countries suffer from PPD [23]. As the consequences of PPD are diverse, including enormous suffering and disabilities [24], a decrease in maternal confidence, and difficulty in responding to the child’s needs, which can affect the child’s mental and physical development [25], it is therefore vital to explore modifiable or controllable factors that can alleviate the risks of PPD.

This study did not find any significant associations between PPD and the type of delivery, delivery complications, baby’s gender, being the first child, social support, or previous depression diagnosis. However, psychosocial factors, especially stress and social support, are well-documented predictors of PPD in the literature. They play a crucial role in preventing any psychological manifestations that may develop during or after delivery. For instance, a cross-sectional study that was conducted in Riyadh, Saudi Arabia, in 2020 found a high prevalence of PPD among those who had recently experienced stressful life events, who had an unsupportive spouse, or who had a CS delivery [11]. Another study, which was conducted in Jeddah city in Saudi Arabia in 2021, found that a history of previous depression, experience with difficult life events, and specific attitudes toward pregnancy were all significantly associated with PPD [12]. Additionally, a study from the Obstetrics ward in Saveetha Medical College and Hospital in India concluded that a lack of social support, low socio-economic status, and family disharmony were significantly associated with PPD [17]. A study conducted in Pakistan in 2021 found several risk factors for PPD, including a previous history of depression, an infant with anomalies, an unplanned pregnancy, gestational diabetes, and any comorbidity in the mother [26]. A study conducted in Ethiopia showed similar results, identifying several factors that were strongly linked with PPD, including premature babies, poor support systems, poor satisfaction with medical care, recurrent verbal abuse, accidents, and the death of a loved one. Other factors such as CS, episiotomy, neonate illness, desired newborn gender, hypertension, and hyperemesis have also been found to be significantly associated with PPD [27]. The present study is limited to women who attended KKUH, which could explain the inconsistency between our findings and the outcomes of the previous studies.

As mentioned previously, the gender of the baby and whether he or she was the mother's first child were not found to be significant in our study. Other studies locally and internationally also found that baby gender was not significantly associated with PPD [27,28].

In this study, we did not find any significant association between mode of delivery and PPD. Many studies found that women who underwent CS were at a higher risk of developing PPD than those who had a spontaneous vaginal delivery. For instance, a research group at the University of Medicine and Pharmacy and Emergency Clinical Hospital in Timisoara, Romania, found that CS, as a mode of delivery, was significantly related to PPD [29]. Another study, which was conducted at Obstetrics Hospital in Egypt, found that induction and augmentation of delivery, delivery before 38 weeks of gestation, and delivery by CS were statistically more common in women suffering from PPD symptoms [14]. We believe that two factors may have played a role in the inconsistency between our results and the outcomes of the previously mentioned studies. First, our study is limited to women who attended KKUH. Second, in the aforementioned study in Romania, women were interviewed two to four days into the postpartum period, which is considered early enough for PPD to be confused with postpartum blues (which refers to mood variations that are common in the first few days after delivery and typically resolve within two weeks without any intervention, unlike PPD) [30].

As mentioned previously, no association between mode of delivery and PPD was found in our study. Other studies found that women who underwent spontaneous vaginal delivery were at a higher risk of developing PPD. The previously mentioned study from the Obstetrics ward in Saveetha Medical College and Hospital in India found that women who had a vaginal delivery rather than a CS had a higher prevalence of PPD [17]. A study conducted in Al-Madinah city in Saudi Arabia in 2021 found that women who delivered spontaneously through a vaginal route had an almost three times greater risk of developing PPD than those who delivered via CS. The same study also found that the prevalence of PPD was particularly high among younger mothers compared to older mothers [19]. The results in the previous studies were justified by the fact that spontaneous vaginal delivery is more painful than CS and that women who delivered vaginally returned home earlier, therefore having to do household chores and getting minimum rest, making them more susceptible to PPD. Our study was limited to only KKUH, whereas the previously mentioned studies involved multiple primary health care centers. For instance, the study that was conducted at Saveetha Medical College and Hospital included women who delivered at three different locations: a college hospital, a primary health center, and at home [17].

Depressive symptoms before or during pregnancy such as frequent grief spells, frustration or anxiety, loss of interest in daily activities, and sleep disturbances were found to significantly increase the risk of PPD in the participants of our study, and this finding is consistent with one study in the literature [31]. In comparison, feelings of guilt, mood swings, a lack of weight gain, or the absence of any symptoms were all found to be insignificant in predicting PPD. Grief in particular can be unwelcome during pregnancy, which is often considered a joyous time. It is also associated with a list of symptoms that tend to be unpleasant [32]. In our study, 59.6% of the participants who had gone through bouts of sadness during or before their pregnancy were more likely to have PPD, compared to another study in the literature that found 11.74% of the participants (n=247) with high EPDS scores experienced maternal anxiety and depression during pregnancy [28].

One of our study’s strengths is that it was able to identify the prevalence of PPD using the validated EPDS scale. Another strength is that the study incorporated multiple predictors of PPD, such as maternal age, family support, symptoms of depression before or during pregnancy, gender of the baby, the method of delivery, whether the baby is the first born, issues during or after birth, and a history of clinically diagnosed depression. We interviewed the mothers in two stages; if they scored less than 9 in the first stage using the EPDS, they were interviewed for a second time. We believed that by using this method, the mothers would be better able to recall their experiences and that we would not miss signs of PPD if they appeared during the first month of delivery.

Limitations and future directions

This study has certain limitations. First, we conducted the study at one center, namely KKUH. Although this center is a tertiary hospital, conducting the study at a larger scale (including several centers and cities) will yield more generalizable results. Indeed, the inter-collaboration between different entities is needed to achieve this goal. Moreover, such a proposed collaboration may earn a higher sample size and more meaningful results. Another advantage is that it will help enroll more representative samples, including some minority groups, which was not feasible for us. Another area for improvement of our study is related to its nature as a descriptive cross-sectional study, which has some inherent disadvantages. Conducting the research using a different methodology, such as a cohort or randomized controlled trial design, could help. Another limitation was that some of the possible PPD predictors were not examined (e.g., the effect of unplanned pregnancy, breastfeeding, socioeconomic status, and some sociodemographic factors). Future studies could consider investigating such factors. Lastly, one unexpected challenge we faced was the COVID-19 pandemic, in which the lockdown with restrictions prevented us from accessing the patients quickly and created a gap during the data collection.

In addition to what was mentioned earlier, we recommend the following. First, patients’ education is crucial to achieving favorable outcomes and avoiding the negative consequences of PPD. Conducting educational campaigns to increase public awareness of PPD could help. Collaboration between obstetrics and gynecology and psychiatry departments could also contribute to achieving such a positive outcome. Another recommendation is screening women with a higher risk for PPD, such as those with a prior history of it. One means to achieve this could be to ensure close monitoring throughout the pregnancy and post-natal and connect them to the psychiatric clinic if needed. Finally, having more support groups and community services could be of great value for those in need and those struggling with PPD. Moreover, we recommend that subsequent studies focus on exploring other factors that were not examined in the current study, such as education level, employment, financial status, number of pregnancies and abortions, breastfeeding, maternal comorbidities, and newborn health, noting that these factors may affect the mother emotionally and could play a role in developing PPD.

## Conclusions

This study aimed to identify the prevalence of PPD and its associated risk factors among women giving birth in KKUH. The results conclude that the prevalence of PPD among the participants is high, with a significant association between PPD and depressive symptoms before or during pregnancy. These findings highlight the importance of screening pregnant women for PPD and linking them to psychiatric services to provide them with proper evaluation, diagnosis, and management. Additionally, we propose that obstetricians and gynecologists provide couples with better PPD awareness and educate them regarding the importance of support to reduce the prevalence of PPD.
